# Supplementary material for: Multiscale light-sheet organoid imaging framework
Source: Nat Commun. 2022 Aug 18;13:4864. doi: 10.1038/s41467-022-32465-z (PMC9388485; doi:10.1038/s41467-022-32465-z)
Supplement: Supplementary file 2 — Reporting Summary [file 41467_2022_32465_MOESM2_ESM.pdf]

nature portfolio

Corresponding author(s): Prisca Liberali

Last updated by author(s): Jul 15, 2022

Reporting Summary

Nature Portfolio wishes to improve the reproducibility of the work that we publish. This form provides structure for consistency and transparency in reporting. For further information on Nature Portfolio policies, see our Editorial Policies and the Editorial Policy Checklist.

Statistics

For all statistical analyses, confirm that the following items are present in the figure legend, table legend, main text, or Methods section.

n/a	Confirmed	
☐	☒	The exact sample size (*n*) for each experimental group/condition, given as a discrete number and unit of measurement
☐	☒	A statement on whether measurements were taken from distinct samples or whether the same sample was measured repeatedly
☒	☐	The statistical test(s) used AND whether they are one- or two-sided<br>*Only common tests should be described solely by name; describe more complex techniques in the Methods section.*
☒	☐	A description of all covariates tested
☒	☐	A description of any assumptions or corrections, such as tests of normality and adjustment for multiple comparisons
☐	☒	A full description of the statistical parameters including central tendency (e.g. means) or other basic estimates (e.g. regression coefficient) AND variation (e.g. standard deviation) or associated estimates of uncertainty (e.g. confidence intervals)
☒	☐	For null hypothesis testing, the test statistic (e.g. *F*, *t*, *r*) with confidence intervals, effect sizes, degrees of freedom and *P* value noted<br>*Give P values as exact values whenever suitable.*
☒	☐	For Bayesian analysis, information on the choice of priors and Markov chain Monte Carlo settings
☒	☐	For hierarchical and complex designs, identification of the appropriate level for tests and full reporting of outcomes
☒	☐	Estimates of effect sizes (e.g. Cohen's *d*, Pearson's *r*), indicating how they were calculated

Our web collection on statistics for biologists contains articles on many of the points above.

Software and code

Policy information about availability of computer code

Data collection	Huygens compute engine 20.10.1p1 64b was used to distill PSF from bead images. Segmentation data collection was obtained with custom code, available in https://github.com/fmi-basel/LSTree.
Data analysis	ImageJ v.1.53h and Paraview 5.8.0 , were used for visualization and analysis of images, Elastix v4.900 for the registration of images. LSTree: https://github.com/fmi-basel/LSTree was used for all other quantifications.

For manuscripts utilizing custom algorithms or software that are central to the research but not yet described in published literature, software must be made available to editors and reviewers. We strongly encourage code deposition in a community repository (e.g. GitHub). See the Nature Portfolio guidelines for submitting code & software for further information.

Data

Policy information about availability of data

All manuscripts must include a data availability statement. This statement should provide the following information, where applicable:

- Accession codes, unique identifiers, or web links for publicly available datasets
- A description of any restrictions on data availability
- For clinical datasets or third party data, please ensure that the statement adheres to our policy

The datasets generated during and/or analysed during the current study are available on reasonable request.

Human research participants

Policy information about studies involving human research participants and Sex and Gender in Research.

Reporting on sex and gender	Not applicable
Population characteristics	Not applicable
Recruitment	Not applicable
Ethics oversight	Not applicable

Note that full information on the approval of the study protocol must also be provided in the manuscript.

Field-specific reporting

Please select the one below that is the best fit for your research. If you are not sure, read the appropriate sections before making your selection.

- [x] Life sciences
- [ ] Behavioural & social sciences
- [ ] Ecological, evolutionary & environmental sciences

For a reference copy of the document with all sections, see nature.com/documents/nr-reporting-summary-flat.pdf

Life sciences study design

All studies must disclose on these points even when the disclosure is negative.

Sample size	No particular statistical method was used to define sample size. Sample size was determined based on previous related studies in the field [Strnad et al Nat Meth (2015), Alladin et al eLife (2020), de Medeiros, Norlin et al Nat Comm (2015), Hof et al BMC Biology (2021)]. For long-term live imaging experiments we assumed that the amount of timepoints comprised in the 7 main different datasets +2 extra backtracking datasets would be sufficient to test the framework.<br>In addition, 12 other datasets from previous publication (Strnad et al, Nature Methods (2015) were used for further challenging the analysis framework)
Data exclusions	No data was excluded.
Replication	Imaging data stem from 4 different experiments. All attempts at replication were successful.
Randomization	Samples were randomly assigned.
Blinding	Same investigators performed data collection and analysis, therefore no blinding.

Reporting for specific materials, systems and methods

We require information from authors about some types of materials, experimental systems and methods used in many studies. Here, indicate whether each material, system or method listed is relevant to your study. If you are not sure if a list item applies to your research, read the appropriate section before selecting a response.

Materials & experimental systems

n/a	Involved in the study	
[ ]	[x]	Antibodies
[x]	[ ]	Eukaryotic cell lines
[x]	[ ]	Palaeontology and archaeology
[ ]	[x]	Animals and other organisms
[x]	[ ]	Clinical data
[x]	[ ]	Dual use research of concern

Methods

n/a	Involved in the study	
[x]	[ ]	ChIP-seq
[ ]	[x]	Flow cytometry
[x]	[ ]	MRI-based neuroimaging

Antibodies

Antibodies used	- anti-DLL1(R&D Systems, # AF3970)<br>- anti-Lysozyme (Dako, # A0099, polyclonal)<br>- anti-E-Cadherin (BD Biosciences, # 610182 Clone 36/E-Cadherin (RUO))

- anti-Limk1 (Abcam, # ab194798, ploYclonal)
- anti-Yap1 (Cell Signaling, # 14074, monoclonal)
- Alexa Fluor 488 Donkey anti-sheep IgG (Thermo fisher scientific A-11015)
- Alexa Fluor 488 Donkey anti rabbit, IgG (Thermo Fisher Scientific; A-21202)
- Alexa Fluor 647 Donkey anti-rabbit IgG (Thermo fisher scientific A-31573)
- Alexa Fluor 568 Donkey anti-mouse, IgG (Thermo Fisher Scientific; A10042)

Validation	Validation statements available from manufacturers:<br>- anti-DLL1(R&D Systems, # AF3970) - https://www.rndsystems.com/products/mouse-rat-dll1-antibody_af3970<br>- anti-Lysozyme (Dako, # A0099, polyclonal) - https://www.agilent.com/en/product/immunohistochemistry/antibodies-controls/primary-antibodies/lysozyme-ec-3-2-1-17-(concentrate)-76124<br>- anti-E-Cadherin (BD Biosciences, # 610182 Clone 36/E-Cadherin (RUO)) - https://www.bdbiosciences.com/eu/applications/research/stem-cell-research/cancer-research/human/purified-mouse-anti-e-cadherin-36e-cadherin/p/610182<br>- anti-Limk1 (Abcam, # ab194798, ploYclonal) - https://www.abcam.com/lim-kinase-1-phospho-t508-antibody-ab194798.html?productWallTab=ShowAll<br>- anti-Yap1 (Cell Signaling, # 14074, monoclonal) - https://www.cellsignal.com/products/primary-antibodies/yap-d8h1x-xp-rabbit-mab/14074?_=1655889309187&Ntt=14074&tahead=true

Animals and other research organisms

Policy information about studies involving animals; ARRIVE guidelines recommended for reporting animal research, and Sex and Gender in Research

Laboratory animals	For C57BL/6 wild type (Charles River Laboratories) which were used for the time-course experiments, 12 weeks old male and 8 weeks old female mice were used. All other lines used were received already as intestines from collaborators. Regarding husbandry, all mice have a 12/12 hours day/night cycle. Medium temperature is 22°C and relative humidity is at 50% .<br>Other mouse lines: H2B-mCherry C57BL/6 x C3H (F1 female intestines heterozygous for H2B-mCherry as kind gift from T. Hiiragi laboratory, EMBL), B6/N x R26 Fucci2 (Tg/+) intestines (kind gift from J. Skotheim lab, Stanford), Lats1Δ/Δ; Lats2Δ/Δ (LATS DKO, intestines kind gift from Jeff Wrana, Department of Molecular Genetics, University of Toronto, Canada).
Wild animals	This study did not involve wild animals
Reporting on sex	Sex was not considered in study design nor in any methods used.
Field-collected samples	study did not involve Field-collected samples
Ethics oversight	Approved by Basel Cantonal Veterinary Authorities and conducted in accordance with the Guide for Care and Use of Laboratory Animals.

Note that full information on the approval of the study protocol must also be provided in the manuscript.

Flow Cytometry

Plots

Confirm that:

- [x] The axis labels state the marker and fluorochrome used (e.g. CD4-FITC).
- [x] The axis scales are clearly visible. Include numbers along axes only for bottom left plot of group (a 'group' is an analysis of identical markers).
- [x] All plots are contour plots with outliers or pseudocolor plots.
- [x] A numerical value for number of cells or percentage (with statistics) is provided.

Methodology

Sample preparation	Sample preparation starts from grown organoids. Transfer of grown organoids into falcon tube, centrifuge and remove remaining supernatant, add Tryple for producing single cells with subsequent washing out of Trple as supernatant from another round of centrifugation, resuspending the pellets with single cells with phenol-free DMEM-F12 with PenStrap and Rock Inhibitor. Filtering the suspension through 30 μm filters and dipensing the cells into the FACS tube directly.
Instrument	BD FACSAria III and BD Influx System
Software	BD Influx System: BD FACS Sortware 1.2.0.142<br>BD FACSAria III: BD FACSDiva Software Version 8.0.1
Cell population abundance	Abundance of sorted cells is given directly after sorting as each of the describe instruments inherently performs single cell counting and provides corresponding statistics. Whenever necessary, purity of the samples is done at the microscope to check for fluorescence intensity.
Gating strategy	First gating based on forward and side Scattering in order to discard debris. Then gating on FSC VS FSC width and SSC VS SSC width are made to avoid getting doublets. Finally, gating on fluorescence VS FSC area is performed to choose the appropriate

marker intensity.

☒ Tick this box to confirm that a figure exemplifying the gating strategy is provided in the Supplementary Information.
