# Supplementary material for: Multiscale light-sheet organoid imaging framework
Source: Nat Commun. 2022 Aug 18;13:4864. doi: 10.1038/s41467-022-32465-z (PMC9388485; doi:10.1038/s41467-022-32465-z)
Supplement: Supplementary file 4 — Description of Additional Supplementary Files [file 41467_2022_32465_MOESM4_ESM.pdf]

Title: Supplementary Software

Description: 1) elephant_mouse_models: contains the linking and spot models trained for tracking prediction for mouse data from Strnad et al. Nature Methods (2015) using the Elephant Tracker

2) lstree_mouse_models: contains the lstree based nuclei segmentation and tracking prediction models for mouse data from Strnad et al. Nature Methods (2015)

3) Elastix_parameters_Affine.txt: parameters file to be used with elastix for registration of fixed organoids to their last position during live imaging

4) parallel_image_compressor.py: python script for compressing images in parallel using the lzw lossless standard

5) rescale_uint8-uint16.ipynb: jupyter notebook for rescaling 8bit into 16bit images during the pre-processing for the mouse data from Strnad et al. Nature Methods (2015)

6) Stardist_config.json: configuration file containing parameters used for nuclei segmentation prediction using Stardist

Title: Supplementary Movie 1

Description: Intestinal Organoid Development. Maximum intensity projection of main datasets used with our framework. On the first column we have two Enterocysts and on the last row the cyst used for fixation and backtracking. All other 4 organoids show a budding phenotype. Not all of the recordings reach 110 hours, and all of them are showing H2B-mCherry (gray) / mem9-GFP (red).

Title: Supplementary Movie 2

Description: Principle of the Digital Organoid Visualizer. On the left the lineage tree overlaid with nuclei volume, on the right nuclei and cell meshes with nuclei color coded as the lineage tree. Cross section of denoised and deconvolved nuclei and membrane data shows very good overlap between segmentation and original recording.

Title: Supplementary Movie 3

Description: Backtracking nuclei of interest. Denoised and deconvolved maximum intensity projection alongside nuclei meshes highlighting backtracked nuclei as in **Figure 4c.** Note that meshes have been rotated in space so that backtracked cells are always clearly visible.

Title: Supplementary Movie 4

Description: Fate of merged nuclei progeny. Example movie of merge progeny cells ending in a growing bud and being discarded into the lumen. Movie corresponds to still images in **Figure 5g,** with H2B-mCherry (gray) / mem9-GFP (red).
